# A mapping survey of digital clinical consultations in maternity care in England

**DOI:** 10.1371/journal.pdig.0000944

**Published:** 2025-07-21

**Authors:** Georgia Clancy, Kerry Evans, Helen Spiby, Victoria Barrett, Candice Sunney, Catrin Evans

**Affiliations:** 1 School of Health Sciences, University of Nottingham, Nottingham, United Kingdom; 2 Nottingham Maternity Research Network, Nottingham, United Kingdom; Iran University of Medical Sciences, IRAN, ISLAMIC REPUBLIC OF

## Abstract

Many areas of healthcare are exploring the use of digital technologies with the aim of improving and expanding care to service users. In England, maternity care is currently undergoing a digital transformation in line with the National Health Service’s (NHS) Long Term Plan, which seeks to enhance care delivery and accessibility through digital tools. However, there is a lack of data on the current use and practice of digital consultations across the country. This project aimed to map and explore how digital clinical consultations are currently being used by NHS maternity care services in England. An online survey was designed to capture data on current practice, guidance and procedures to address potential inequalities. The survey was distributed to each NHS Trust (n = 121) that provides maternity care (specifically to senior maternity care professionals and digital maternity leaders who could provide an overview of how digital consultations were being used where they worked). The survey was open between January and March 2024. 53 completed surveys were received representing 39 different organisations (32% of those currently providing maternity care in England). Quantitative summary statistics indicated that telephone consultations were the most commonly used digital modality across all stages of the maternity care pathway. Thematic analysis identified barriers such as a lack of staff consultation and lack of staff training on the use of digital consultations. It was uncommon for women to be asked about their consultation preferences or assessed for individual needs. In conclusion, the findings reveal significant variation in the use of digital consultations, highlighting a gap between policy intentions and practice. Key areas for improvement in the delivery and implementation of digital consultations include staff training, systems to record women’s consultation preferences/needs, and more research to support digital inclusion. The findings of this survey have the potential to have applications beyond maternity care and in different geographical contexts.

## Author summary

In recent years there has been a digital transformation in maternity care in England which includes the roll-out of digital consultations. However, the extent to which digital consultations are being used in practice, how they are being used and the nature of any training or guidance that is in place to support this, is currently unclear. To answer these questions, an online survey was distributed to senior maternity care professionals and digital maternity leaders who could provide an overview of how digital consultations were being used where they worked. Findings reflected variation in the use and practice of digital consultations in maternity care across England. Telephone consultations were found to be the most commonly used type of digital consultation (compared to video call, messaging services, email and App). Responses indicated low levels of staff training. Likewise, relatively few organisations reported a systematic approach to recording of women’s preferences or needs concerning the use of digital consultations in their care. The findings of this survey can be used to support the digital transformation of maternity care currently taking place in England.

## Introduction

In the UK, digital transformation is a key part of the National Health Service’s (NHS) Long Term Plan and the Maternity Transformation Programme in England [1,2]. Digitally-linked and enabled services are proposed as a key mechanism for supporting improvements in the provision of safe and personalised care, as envisioned by the NHS England maternity care policy, ‘Better Births’ [3]. This policy imperative has been supported by the Royal College of Midwives’ (RCM) (2021) ‘Position Statement on Digital Technology in Maternity Care’ to facilitate the pursuit of three primary objectives: (i) to improve the experience of women using maternity services; (ii) to enhance the safety and quality of maternity care, and (iii) to support midwifery practice and free up midwifery time to improve clinical care [4]. This paper focusses on exploring current practices and concerns regarding digital consultations in maternity services in England.

The digital transformation in NHS maternity care is being led through local, regional and national networks of ‘digital midwives’ and digital leads in obstetrics. For service users, this includes the roll out of features such as digital/remote consultations, digital maternity records, messaging systems, online antenatal classes, websites for local services, and remote monitoring systems (to support the management of conditions such as gestational diabetes or hypertensive disorders). Although all NHS maternity services are on a pathway to digital transformation, there is currently considerable variation in the digital maturity and implementation of digital services between and within NHS Trusts (local NHS organisations which deliver services for a specific geographical area or function) and Integrated Care Systems (partnerships between NHS services and local partners such as the voluntary sector, local government or social care providers) [5,6].

Alongside the digital transformation in maternity care in England is an urgent need to address inequalities in women’s experiences and poorer clinical outcomes linked to race, ethnicity, migration status and social deprivation [7,8]. As digital developments continue, it is vital to ensure that new systems and practices are inclusive and can support equity objectives [9–12]. However, the mechanisms through which existing pathways of inequality intersect with digital care are still relatively unknown [13–15].

This paper presents the results of a mapping survey that aimed to illuminate how digital consultations are currently being used by NHS maternity care services across England. The survey follows-on from a realist evidence synthesis – the ARM@DA project (A Realist inquiry into Maternity care @ a DistAnce) – that investigated “what works, for whom and in what context” in relation to the implementation of digital clinical consultations (synchronous telephone and video appointments) in UK maternity care [16,17]. The evidence review was conducted with input from diverse healthcare professional and service user stakeholder groups, and proposed a set of programme theories and implementation principles that could be applied across diverse maternity settings [18]. A key finding from the evidence synthesis highlighted challenges for staff in accessing digital resources, devices and internet connectivity whilst working onsite at NHS facilities, as well as when providing offsite community-based care, including in women’s homes [19]. To enable the safe and high-quality incorporation of digital consultations into practice, as well as provider acceptance and confidence, it was also found that staff needed training and ongoing support on a broad range of aspects related to digital care [20]. This included how to use the technology and software facilitating digital consultations, how to communicate effectively with service users at a distance and how to assess women’s clinical needs remotely [21].

Other key findings of the realist synthesis related to service user preferences and needs around digital consultations. For example, women’s preferences in relation to consultation modality were highly variable as a result of individual dispositions, needs, skills, resources and lifestyles. The review suggested that asking women about their consultation preferences and life circumstances at the start of their maternity journey could provide an element of control and personalisation. Another key review finding was the need to attend carefully to digital inclusion – making sure that remote consultations did not create additional barriers to access or uptake of maternity care. For example, it was considered important to assess any potential communication barriers (language, hearing, neurodiversity), safeguarding and privacy concerns (e.g., whether a woman had access to her own phone and/or could talk privately) or other issues (e.g., social anxiety), as well as digital literacy (women’s ability to understand and navigate remote care) and access to digital resources (including phones or the internet). These issues were identified as particularly salient for some groups of women (e.g., refugees/asylum seekers) for whom digital services were identified as potentially complicating an already confusing terrain of service access points, leading to new and additional complexities in navigating care and therefore, to potential delays in accessing the right services or information.

The evidence synthesis concluded that: (i) there is significant variability in the use of digital consultations across NHS maternity care, (ii) there is a lack of information about the current use of digital consultations in maternity care, and (iii) there is a lack of information on local guidance being used to inform good practice. There is particular concern that remote consultations have the potential to exacerbate health inequalities, but there is a lack of data on how this is being addressed in practice. Similar concerns have been raised in studies and reviews worldwide [22–29]. This mapping survey sought to address these information gaps.

Whilst this paper focuses on maternity care in England, digital consultations in different modalities are becoming increasingly common in healthcare services around the world, a shift that was expedited during the COVID-19 pandemic when many services were forced to move away from in-person care [24,30–33]. As such the findings reported here may be applicable to a range of geographical contexts and healthcare settings.

### Aim

To map and explore how digital clinical consultations are currently being used by NHS maternity care services in England.

### Objectives

1. To explore the extent, and for which clinical situations, digital clinical consultations are currently being used by maternity services in NHS England.
2. To investigate what technological modalities are being used for digital clinical consultations.
3. To understand what local guidance, training and systems are in place to support the use of digital clinical consultations.
4. To explore what provision, if any, is in place to address inequalities/inclusion in the use of digital clinical consultations.

## Materials and methods

Ethical approval for this study was granted by the Faculty of Medicine and Health Sciences Research Ethics Committee at the University of Nottingham, Ref: FMHS 37-1023.

An online survey comprising of 48 questions split into eight sections was developed in a secure and GDPR compliant, MS Forms (see S1 File). The survey was developed to address information gaps identified in the aforementioned realist evidence synthesis and to provide a clearer picture of how remote consultations are being implemented [16]. As such a bespoke survey was created to map current practices of digital consultations at a particular point in time. The aim was not to develop a measurement tool. Questions were developed through a rigorous, iterative and collaborative process involving the research team (comprised of a service user representative, midwives, obstetrician, sociologist and health services researcher) and consultation with the project advisory group (comprised of representatives from the RCM, Royal College of Obstetricians [RCOG] and NHS Transformation Directorate). The survey consisted of single- and multiple-choice questions, Likert-scales and free-text questions. Prior to dissemination, the survey was independently piloted by three midwives and three obstetricians (some with a digital remit), and their feedback led to small changes which included clarifying language and improving survey navigation. The survey development process with key constituencies provided expert consensus and ensured both content and construct validity. It was not deemed appropriate to undertake a test-retest assessment for reliability as this would have required busy clinicians to complete the survey twice. No concerns about inter-rater reliability were raised during the development or piloting.

A link to the online participant information sheet was included in the introduction to the survey and informed consent was obtained within the survey itself. A purposive sampling strategy, assisted by snowball sampling, was used to target senior maternity care professionals (e.g., directors/heads of midwifery, heads of obstetrics, consultant midwives, consultant obstetricians) and digital maternity leaders (e.g., digital midwives, digital obstetricians) who had in-depth knowledge of how digital consultations were being used in their work places. The aim was for the survey to represent as many of the NHS Trusts in England providing maternity care as the denominator (rather than calculating a sample of individuals working within the Trusts) [34]. At the time of the survey this was 121 NHS Trusts. The survey link was distributed via professional bodies (e.g., RCM and RCOG), professional networks (NHS digital maternity leaders, British Intrapartum Care Society) and on social media. Reponses were anonymous and minimal identifiable information was collected such as the respondent’s role/position (to confirm eligibility) and the name of the NHS Trust in which they worked (to keep track of which NHS Trusts has completed the survey). However this data was solely for monitoring purposes, was kept separate from the responses regarding digital consultations and could only be accessed by the study team. The research team anticipated that multiple participation was unlikely to occur as healthcare professionals are often very busy and there was no financial incentive for taking part. The survey was open for six weeks between January-March 2024.

The data collected in MS Forms was downloaded onto an Excel spreadsheet and free-text responses were copied into a Word document. The quantitative data was ‘cleaned’ and analysed in Excel using descriptive statistics. A descriptive thematic approach was taken for analysis of the qualitative data in NVivo12. This involved an iterative process of line-by-line coding in which data was mapped onto sub-themes and then final themes, alongside team discussion [35,36]. The data for some qualitative questions was quantified during coding in NVivo to identify the most frequent answers in the sample. Additionally, the data for questions 46–48 was analysed together and quantified due to the overlap in participant responses across these three questions (see S3 File). This paper reports the survey in accordance with the CROSS reporting checklist for survey studies (see S2 File) [37].

## Results

54 completed surveys were received from individuals representing 39 different NHS Trusts (out of a potential 121). One survey was removed as the participant was not a senior maternity care professional or digital maternity leader and therefore ineligible. Final data represented insights from 32% of NHS Trusts in England that currently provide maternity care (39/121), with responses received from all geographical regions in England, reflecting varying patterns of maternity care across the country. Full respondent information can be found in Table 1. 79% of respondents were midwives (including 47.2% digital midwives) and 21% were obstetricians (including 3.8% digital leads for obstetrics).

**Table 1:** Respondent information.

	Frequency	Percent (%)
**Region**		
East Midlands	4	7.5
East of England	13	24.5
Greater London	8	15.1
North East	3	5.7
North West	5	9.4
South East	5	9.4
South West	3	5.7
West Midlands	8	15.1
Yorkshire and the Humber	4	7.5
**Maternity service configuration**		
Single site	27	51
Multiple site	26	49
**Births supported per year**		
Less than 2,500	4	8
2,500 - 4,000	12	23
4,000 - 5,000	13	25
5,000 - 6,000	7	13
More than 6,000	17	32
**Role/position of respondent**		
Director/Head of midwifery	5	9.4
Midwifery matron	4	7.5
Digital midwife	25	47.2
Consultant midwife	5	9.4
Specialist midwife	3	5.7
Consultant obstetrician	4	7.5
Consultant obstetrician and gynaecologist	5	9.4
Digital lead for obstetrics	2	3.8

### Consultation modalities across the maternity pathway

**Fig 1:**
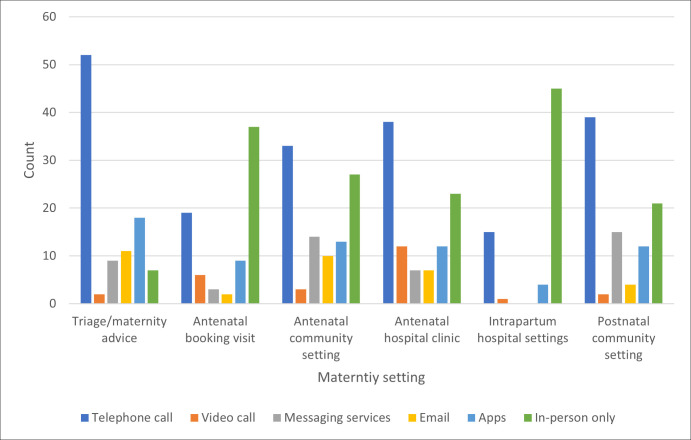
Distribution of consultation modalities across the maternity pathway.

Respondents were asked about the different modalities (telephone call, video call, messaging services, email, Apps and in-person) that were used to conduct consultations across different parts of the maternity care pathway (see Fig 1). 98% of respondents reported that their service used telephone calls for triage/maternity advice, making this the dominant modality used in this setting. Indeed, more respondents said that telephone calls were used for triage than any other combination of consultation modality and clinical context. Across the other five settings that respondents were asked about (antenatal booking visit, antenatal community appointments, antenatal hospital appointments, intrapartum care and postnatal care), telephone calls and in-person care were consistently reported as the main consultation modalities used. Video calls were the least frequently reported.

### Maternity care professional choice and support

It is well known that staff buy-in is an important facilitating factor in the introduction of new approaches to care [16]. The survey found that just 13% of respondents reported that staff in their clinical area had been asked about their preferences for providing digital consultations; 48% said that staff had not been asked and 38% ‘did not know’. When asked if staff were able to ‘opt-out’ of providing digital consultations, the response from maternity leaders was mixed; 29% said ‘yes’ they could opt-out, 26% said ‘no’ they could not, and 36% ‘did not know’.

When asked who in their clinical area was currently providing digital maternity consultations, the leading answers were ‘some midwives’ (40%) and ‘some obstetricians’ (38%). A small number of respondents also highlighted ‘Other’ groups using digital consultations such as specialist midwives, diabetic nurse specialists and anaesthetists, demonstrating the potential for these technologies to be used to support women who need additional care during maternity. The vast majority of respondents (80%) also reported that digital consultations were integrated with face-to-face care, as opposed to holding standalone digital clinics. This suggests that digital consultations are being incorporated flexibly into working practice. Indeed as the qualitative data showed, the increased flexibility and efficiency of digital consultations for staff and services, as an alternative to in-person care, was perceived to be a key benefit for respondents.

Given that digital transformation represents a considerable change to the delivery of care, respondents were asked if their maternity service was following a digital strategy, and if so, who developed this. 94% of respondents stated ‘yes,’ their maternity service did have a digital strategy, with 6% answering that they ‘did not know’. In a free-text box, respondents stated that these strategies were often developed ‘in-house’, Local Maternity and Newborn System(s) or NHS Trust(s). Few responses referred to national policy or strategy. This local focus might explain the variation in the use of digital consultations across the country.

In relation to training to support digital consultations, the most common form of training that respondents reported organisations were providing related to ‘information governance’ (28%) and ‘technical aspects’ of using the technology/software (22%) (see Table 2). However, 9.9% of respondents reported that their organisation did not provide any formal training on digital consultations at all. Interestingly, there seemed to be an observed association between the areas where less training was being provided - assessing clinical need, effective communication and personalising care - and the top three concerns raised by respondents about using digital consultations - lack of physical examination/tests, communication barriers and digital literacy (see S3 File for qualitative data). These findings suggest a need for training in these areas. Potentially compounding the effects of limited training for staff, the survey found that 57% of respondents also reported that their organisation did not have maternity specific guidelines/protocols to follow when conducting digital consultations.

**Table 2:** Organisational training provided for staff conducting digital consultations.

Aspects of training:	Frequency	%
No training	11	9.9
Technical	25	22.5
Information governance	32	28.8
Incorporating digital consultations into practice	3	2.7
Clinical need, assessing safety/risk	15	13.5
Effective communication	10	9.0
Personalisation of care	14	12.6
Other	1	0.9

### Digital resources and connectivity

In terms of the facilities and technologies available to staff when conducting digital consultations, the majority (70%) of respondents said that staff did have dedicated spaces (i.e., somewhere private and quiet) in which consultations could take place. Most respondents also reported that staff used a range of employer-provided digital devices (mobile phones, landline phones, tablets, laptops and desktop computers) for conducting digital consultations both when working on NHS sites and offsite in the community. Just 3% said that staff had to use personal devices on NHS sites, and 4% when working offsite. 70% also said that staff had digital connectivity resources (Wi-Fi/mobile internet data) on NHS sites and offsite, with just 9% reporting that staff used their personal internet/data allowances to enable digital consultations.

**Fig 2:**
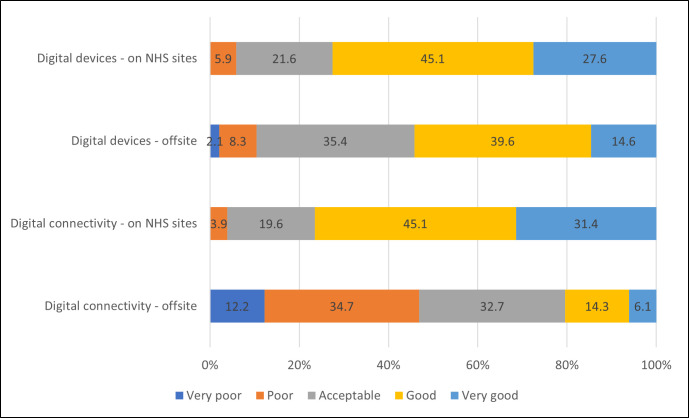
Suitability of digital devices and digital connectivity resources for digital consultations.

As shown in Fig 2, survey participants gave positive responses when asked about the suitability of (i) digital devices on NHS sites (45.1% ‘good’, 27.6% ‘very good’), (ii) digital devices offsite (39.6% ‘good’, 35.4% acceptable) and (iii) digital connectivity on NHS sites (45.1% ‘good’, 31.4% ‘very good’). However, digital connectivity offsite appeared to be more challenging, with 34.7% of respondents rating this as ‘poor’, and 32.7% rating this as ‘acceptable’. This could have a particular impact on community midwives who are most likely to be working offsite and highlights variability in the contemporary nature of the online medical record. This could lead to additional work if staff have to wait to update records when they can reconnect to the internet.

### Women and service users

Respondents were asked if the consultation preferences of women receiving their service were assessed at any point during their maternity care (i.e., if they were happy to have digital consultations, and if so by what modality). As Table 3 shows, just 13.2% of respondents felt that women were routinely asked about their consultation preferences and that this preference was formally recorded in their notes. Furthermore, just 19% of respondents said that women’s preferences were regularly reviewed throughout the maternity care journey, to ensure that care was continuing to be delivered in accordance with the women’s needs, preferences and life circumstances. Indeed, when asked in an open-ended question what benefits respondents perceived from using digital consultations, the most frequent answer was the potential to reduce the burden of care for women/service users. However, knowing and recording women’s consultation preferences is a key part of enabling this.

**Table 3:** Obtaining women/service user consultation preferences.

Obtaining women/service user consultation preferences. I.e. if they are happy to have digital consultations and by what technological modality.	Frequency	%
Yes - formally recorded in notes	7	13.2
Yes - informally, not recorded in notes	15	28.3
No	17	32.1
Don’t know	14	26.4

As Table 4 shows, respondents reported that various aspects of women’s suitability for digital consultations were being assessed in order to support the provision of safe and appropriate care. Whilst ‘language/communication needs’ (18.9%) and ‘clinical need/risk’ (17.6%) were the aspects most commonly reported to be assessed, this data shows that staff in responding organisations were on average assessing 3–4 different aspects of suitability. However, 13% of respondents said that there was no assessment of women’s suitability for digital consultations in their organisation.

**Table 4:** Assessment of suitability for digital consultations.

Areas of assessment for women/service user suitability for digital consultations	Frequency	%
No assessment	19	12.8
Clinical need/risk (including when an in-person assessment is needed)	26	17.6
Access to digital devices/connectivity and digital literacy	23	15.5
Language/communication needs	28	18.9
Psychosocial status (e.g., mental health conditions)	23	15.5
Safeguarding concerns	25	16.9
Don’t know	1	0.7
N/A	3	2.0

Nearly 34% of respondents said that women were given no additional information or support to help them access digital consultations. Where information was provided, it was most often reported to be verbal (26%), rather that printed (15%), digital (11%) or video (5%). This could be problematic for women with language or communication difficulties who might struggle to understand information verbally. It also means that women would not have information to refer back to at a later date. Since service user access to digital devices and resources is key to the uptake of digital consultations, respondents were asked whether their organisations had policies and procedures in place to support equitable access. Just 13% said ‘yes’ - including the provision of free devices/internet data, interpreters and translated resources - 33% said ‘no’ and 54% ‘did not know’.

Full results from the mapping survey into the use of digital consultations in maternity care can be found in S3 File.

## Discussion

Telephone calls and in-person care were reported to be the two most consistently used digital consultation modalities utilised across the maternity care pathway in England. This is perhaps unsurprising given that these modalities have been a common part of healthcare practice in the UK for years, whilst other modalities such as video calls, messaging services, email and Apps have been introduced much more recently. Indeed, the realist review that preceded this survey found that telephone calls tended to be the default option used by staff when face-to-face care was not possible since newer digital modalities (i.e., video calls or Apps) could present additional barriers to use [16,17,38,39]. However, digital transformation became a focus of NHS maternity care following the publication of the National Maternity Review Better Births policy report in 2016, and has since been reiterated in the subsequent Harnessing Digital Technology workstream (2021) and Three year delivery plan for maternity and neonatal services (2023) document [2,40,41]. This repetition over many years raises questions about the speed and extent to which the digital transformation is happening and suggests a potential gap between policy and practice. Indeed the recent Independent Investigation of the National Health Service (2024) noted that over the last decade there have been missed opportunities to incorporate digital technologies into NHS which are central to the organisation’s future, highlighting the slow uptake [42].

Although telephone calls have been used in practice for a long-time [43], they have limitations for women and healthcare providers due to the lack of physical examination and non-verbal cues, as well as potentially exacerbating communication difficulties [20,44–46]. The survey showed that telephone continues to be a key consultation modality in NHS maternity care, highlighting the importance of getting telephone calls right so that they can be used safely [43].

The data pertaining to whether or not organisations asked staff about their consultation preferences, as well as their ability to ‘opt-out’ of providing digital consultations was mixed, suggesting some variation in practice between services and teams. However, professional autonomy in the choice of consultation mode to suit the service user’s wants and needs, as well as their own preferences, experience-level and skillset, can be key to improving staff acceptance of digital consultations and sustained uptakes of these new technologies [16,21,47].

The survey results show that organisations may not routinely be providing digital consultation skills training (beyond information governance). This is concerning since the skillset providers have developed for in-person consultations might not translate directly to remote care, impacting staff confidence, uptake and safety [48]. This finding is consistent with the literature in which a lack of training appears to be commonplace [16,46,49] and suggests a potential gap in recognising the unique challenges that digital consultations pose and how professionals may need to adapt their in-person skillset to deliver remote care that is safe, effective and personalised. There was no clear link between different regions or NHS Trusts and the types of training provided. Often there was variation between the responses from participants working in the same regions/NHS Trusts, suggesting that access to, or awareness of, training may vary between staff. Particularly concerning was the lack of training and support for staff in areas that seem to be most important to them, i.e., how to respond when there is a lack of physical examination/tests, how to address communication barriers and how to assess/support digital literacy. Addressing the need for comprehensive training on digital maternity care in policy will be key to driving action and improvement for all staff working in this area. A short e-learning course based on the findings of the preceding realist review has been developed to support this [50]. Such training, particularly focussing on these areas of concern raised by respondents, could be offered at pre-registration level as well as part of professional development for those already in practice.

When it came to ascertaining women’s consultation preferences, assessing their suitability for digital consultations and how they could be supported to access them, the results suggested room for improvement. This is important and should be incorporated into policy because the formal recording of preferences can inform different staff which consultations modalities are suitable for the woman in their care, as well as any arrangements that need to be made in advance of an appointment, such as an interpreter. Just 13% of respondents said that women’s consultation preferences were formally recorded in their notes. Indeed, previous research has indicated that there is a need to develop systems and templates for the recording of consultation preferences, digital access and inclusion needs [16]. Nearly 34% of respondents said that women in their organisations were not given additional information or support specifically to help them access digital consultations. Indeed, a key implementation principle identified in the realist synthesis [17] was that women should be provided with comprehensive information about what to expect from digital care, how it would fit with in-person care, as well as guidance on how to use the technology. Additionally, this information should be available in a wide range of languages and formats to meet diverse needs.

As digital developments in maternity care continue to roll out, it is vital to consider the impact that new systems and practices might have on a diverse range of service users, particularly in light of the current inequalities in maternal outcomes and experiences for women in England [8]. The realist review which preceded this survey identified numerous challenges to access faced by women from disadvantaged groups (e.g., digital literacy/resources, as well as communication barriers and knowledge of care systems), and also identified various mechanisms to improve outcomes [see [16] for more detail on potential inequalities, [17]. In this study, just 13% of respondents stated that their organisations had policies and procedures in place specifically to support access to remote consultations. The high proportion of respondents who said either that their organisation did not have policies to support equitable access (or they did not know if their organisation had policies), suggests that this need is possibly being overlooked and existing inequalities in maternity care could be exacerbated by digitalisation. However, in light of multiple policy directives to address inequalities in maternity care, this picture may also now be changing with various initiatives being introduced to support digital inclusion. For example, a national initiative coordinated by the NHS digital midwives’ leadership team in collaboration with the ‘Good Things Foundation’ was announced earlier this year (February 2024). This initiative aims to make data and devices accessible to any woman in need during pregnancy and the post-partum period. There is an aim to establish digital maternity hubs in each NHS Trust in England which can provide SIM cards and phones and can also signpost women to local digital literacy training where required. Community based ‘digital champions’ have also been proposed to support service navigation and digital skill development [51]. Other approaches related to the use of Apps in maternity care are seeking to improve accessibility and language functionality such as the Mum and Baby App [52] used in many London Trusts, and the JANAM App [53] specifically aimed at the needs and cultural context of South Asian women in Leicester.

The digital transformation of NHS maternity care is a multifaceted issue due to the complexity of the maternity care system, as identified by the preceding realist review [16,17] and latest NHS Maternity Digital Maturity Assessments which explore regional variation in-depth [5,6]. In the long-term, improving the digital maturity of NHS Trusts will be key to reducing the current variability in the use of digital consultations across NHS maternity care to create a more cohesive approach across the country. Learning lessons from the initiatives outlined above to support equitable access and improving staff training nationally is also key.

### Recommendations

The findings of this survey illuminate and support implementation principles identified in the recent realist synthesis of digital consultations in maternity care [16,17], as well as other relevant literature [21,22,46]. The survey suggests a need for training [50] and guidance for staff specific to the digital modalities being used in their workplace. This could include topics such as: assessing clinical need without a physical examination, how to communicate with service users effectively remotely, and how to ensure that remote care is personalised to women’s individual needs (including their digital literacy/resources). Additionally there is a need to develop templates and systems through which maternity care professionals can ascertain and formally record women’s consultation modality preferences and digital accessibility needs.

Since telephone consultations were found to be the leading digital modality currently in use, future research might focus on improving best practice in this area. There is a need for more rigorous and robust research to evaluate different digital consultation modalities (compared against each other as well as compared with in-person care), specifically in relation to clinical and safety outcomes, as well as service user satisfaction. Such research is important to provide better evidence to inform decision-making for design and delivery of services that include remote consultation options. It would also be helpful to further explore how best to support digital inclusion and improve digital connectivity in the community, so that the digital transformation does not exacerbate existing inequalities in maternity care. Finally, it would be important to conduct research with service users to explore the similarities or differences in their views and experiences of digital maternity care, compared with those of professionals.

### Strengths and limitations

A limitation of this survey is the relatively small number of responses. Just under a third of NHS Trusts in England that currently provide maternity care were included in the data. Unfortunately, a low-response rate in surveys is increasingly common, with a steady downward trend in healthcare professionals completing surveys having been reported for some time [54,55]. As such, achieving a response rate of one third for a national survey may also be considered a strength, and indeed the geographical spread of responses which incorporates different patterns of maternity care increases the generalisability of findings. Furthermore, the sampling approach was highly targeted and it is noteworthy that the majority of responses came from midwives or obstetricians with a digital focus. As such, participants are likely to have had good knowledge of how digital consultations were being implemented in their workplace. Thus whilst more precise detail or data is needed, this survey has nonetheless provided a picture showing that (i) there is variation (which needs to be addressed) and (ii) there are common needs for staff training and assessment of women’s preferences.

A further limitation is the amount of detail that can be captured in a survey design. Attempts were made to mediate this by incorporating a number of open-ended questions where respondents could answer in their own words. However, these free-text responses were still often quite brief, perhaps as a consequence of the survey approach or because respondents were busy professionals who did not have time to write lengthy answers. As such these responses sometimes lacked context. For example when asked about their concerns regarding digital consultations, it was not always clear if responses related to professionals, service users or both. Although the survey was developed with extensive stakeholder consultation and a pilot, a limitation is that the survey did not undergo a test-retest. The challenges of trying to engage busy clinicians meant that this additional demand on their time was not considered possible. Indeed, even though the mapping survey did not aim to include full validity and reliability testing at this stage of investigation, a strength is that it has identified important dimension of safe and high-quality digital consultations which can inform the development of future measurement tools.

Finally, a key strength of this project has been the input from different expert stakeholders including a PPI co-applicant (CS), Project Advisory Group with representation from key professional bodies, and piloting by midwives and obstetricians. This helped to improve the relevance of the survey, especially given the variation that exists within NHS maternity care services, between different service contexts and staff roles. However it is important to note that this bespoke mapping survey was created and undertaken for a specific population at a particular point in time and we were not intending to develop a measurement tool generalisable to other populations or contexts

## Conclusions

This survey illuminates the findings and recommendations made in the preceding realist synthesis [16] and other relevant international literature [24,26,29]. The results affirm the importance of ascertaining service user preferences, providing staff training and support, and addressing equitable access to digital services. The survey also appears to confirm that there is variability in the digital transformation of NHS England maternity care services, both in terms of whether or not new consultation technologies are being adopted and if they so, in the ways in which they are being implemented. This results in localised digital strategies and solutions to some of the issues associated with digital care identified here rather than a coherent national approach. This has the potential to create disparity between service users who have different digital maternity care options, choices and support as a result of where they live. With digital transformation set to be a key aspect of maternity and wider healthcare reform in the UK and elsewhere, many of the key findings reported here have the potential to be applicable in a variety of healthcare settings and geographical contexts.

## Supporting information

S1 File Survey on digital clinical consultations in maternity care. (PDF)

S2 File CROSS Checklist for Reporting Of Survey Studies. (DOCX)

S3 File Survey dataset. (DOCX)

## References

[1] NHS England. NHS Long Term Plan. 2019.

[2] NHS England. Maternity Transformation Programme.

[3] National Maternity Review. Better Births: Improving Outcomes of Maternity Services in England: A Five Year Forward View for Maternity Care. 2016.

[4] Royal College of Midwives. Digital Technology in Maternity Care: A Position Statement. 2021.

[5] NHS Digital. Maternity Digital Maturity Assessment (Regional Summary). 2022.

[6] NHS Digital. Maternity DMA Report: Digital Maturity Assessment of Maternity Services in England. 2018.

[7] Knight M, Bunch K, Felker A, Patel R, Kotnis R, Kenyon S, et al. Saving Lives, Improving Mothers’ Care Core Report - Lessons learned to inform maternity care from the UK and Ireland Confidential Enquiries into Maternal Deaths and Morbidity 2019-21. Oxford: University of Oxford, National Perinatal Epidemiology Unit; 2023.

[8] Draper ES, Gallimore ID, Kurinczuk JJ, Kenyon SL, (on behalf of the MBRRACE-UK Collaboration). MBRRACE-UK Perinatal Confidential Enquiry: A comparison of the care of Black and White women who have experienced a stillbirth or neonatal death - State of the Nation Report. Leicester: The Infant Mortality and Morbidity Studies, Department of Population Health Sciences, University of Leicester; 2023.

[9] Hughson J-AP, Daly JO, Woodward-Kron R, Hajek J, Story D. The Rise of Pregnancy Apps and the Implications for Culturally and Linguistically Diverse Women: Narrative Review. JMIR Mhealth Uhealth. 2018;6(11):e189. doi: 10.2196/mhealth.9119 30446483 PMC6269626

[10] The King’s Fund. Ensuring Digitally Enabled Health Care is Equitable and Effective for All. 2023.

[11] NHS England & NHS Improvement. Equity and Equality: Guidance for Local Maternity Systems. 2021.

[12] Rothman K. Widening digital inclusion during pregnancy. Practising Midwife. 2023;26(1):32.

[13] Husain L, Greenhalgh T, Hughes G, Finlay T, Wherton J. Desperately Seeking Intersectionality in Digital Health Disparity Research: Narrative Review to Inform a Richer Theorization of Multiple Disadvantage. J Med Internet Res. 2022;24(12):e42358. doi: 10.2196/42358 36383632 PMC9773024

[14] Veinot TC, Mitchell H, Ancker JS. Good intentions are not enough: how informatics interventions can worsen inequality. J Am Med Inform Assoc. 2018;25(8):1080–8. doi: 10.1093/jamia/ocy052 29788380 PMC7646885

[15] TPXimpact, NHS Race and Health Observatory. Digital apps and reducing ethnic health inequalities. 2023.

[16] Evans C, Clancy G, Evans K, Booth A, Nazmeen B, Sunney C, et al. Optimising digital clinical consultations in maternity care: a realist review and implementation principles. BMJ Open. 2024;14(10):e079153. doi: 10.1136/bmjopen-2023-079153 39486829 PMC11529580

[17] Evans C, Clancy G, Evans K, Booth A, Nazmeen B, Sunney C, et al. How to Implement Digital Clinical Consultations in UK Maternity Care: the ARM@DA Realist Review. NIHR Health and Social Care Delivery Research. 2024. 10.3310/WQFV7425 40417997

[18] Evans C, Clancy G, Evans K, Booth A, Nazmeen B, Timmons S, et al. Developing initial programme theories for a realist synthesis on digital clinical consultations in maternity care: contributions from stakeholder involvement. J Res Nurs. 2024. 10.1177/17449871241226911 PMC11271666 39070565

[19] Cordasco KM, Katzburg JR, Katon JG, Zephyrin LC, Chrystal JG, Yano EM. Care coordination for pregnant veterans: VA’s Maternity Care Coordinator Telephone Care Program. Transl Behav Med. 2018;8(3):419–28. doi: 10.1093/tbm/ibx081 29800406

[20] Borrelli S, Downey J, Fumagalli S, Colciago E, Antonella N, Spiby H. How should a video-call service for early labour be provided? A qualitative study of midwives’ perspectives in the United Kingdom and Italy. Women Birth. 2023. 10.1016/j.wombi.2023.06.006 37365096

[21] Hinton L, Dakin FH, Kuberska K, Boydell N, Willars J, Draycott T, et al. Quality framework for remote antenatal care: qualitative study with women, healthcare professionals and system-level stakeholders. BMJ Quality Safe. 2022;12:12. 10.1136/bmjqs-2021-014329 PMC11041557 35552252

[22] Cantor AG, Jungbauer RM, Totten AM, Tilden EL, Holmes R, Ahmed A, et al. Telehealth Strategies for the Delivery of Maternal Health Care : A Rapid Review. Ann Intern Med. 2022;175(9):1285–97. doi: 10.7326/M22-0737 35878405

[23] Konnyu KJ, Danilack VA, Adam GP, Friedman Peahl A, Cao W, Balk EM. Changes to Prenatal Care Visit Frequency and Telehealth: A Systematic Review of Qualitative Evidence. Obstet Gynecol. 2023. 10.1097/AOG.0000000000005046 36649343

[24] Society for Maternal-Fetal Medicine, Healy A, Davidson C, Allbert J, Bauer S, Toner L, et al. Society for Maternal-Fetal Medicine Special Statement: Telemedicine in Obstetrics - Quality and Safety Considerations. Am J Obstet Gynecol. 2022. 10.1016/j.ajog.2022.12.002 36481188

[25] Wu K, Lopez C, Nichols M. Virtual visits in prenatal care: an integrative review. J Midwifery Women Health. 2021. 10.1111/jmwh.13284 34767317

[26] van den Heuvel JFM, Ayubi S, Franx A, Bekker MN. Home-Based Monitoring and Telemonitoring of Complicated Pregnancies: Nationwide Cross-Sectional Survey of Current Practice in the Netherlands. JMIR Mhealth Uhealth. 2020;8(10):e18966. doi: 10.2196/18966 33112250 PMC7657725

[27] Palmer KR, Tanner M, Davies-Tuck M, Rindt A, Papacostas K, Giles ML, et al. Widespread implementation of a low-cost telehealth service in the delivery of antenatal care during the COVID-19 pandemic: an interrupted time-series analysis. Lancet. 2021;398(10294):41–52. doi: 10.1016/S0140-6736(21)00668-1 34217399 PMC8248925

[28] Balk E, Konnyu K, Cao W, Reddy B, Danilack V, Adam G, et al. Schedule of Visits and Televisits for Routine Antenatal Care: A Systematic Review. Comparative Effectiveness Review No. 257. AHRQ Publication No. 22-EHC031: Brown Evidence-based Practice Center. 2022. 35862565

[29] DeNicola N, Grossman D, Marko K, Sonalkar S, Butler Tobah YS, Ganju N, et al. Telehealth Interventions to Improve Obstetric and Gynecologic Health Outcomes: A Systematic Review. Obstet Gynecol. 2020;135(2):371–82. doi: 10.1097/AOG.0000000000003646 31977782 PMC7012339

[30] Kabongo EM, Mukumbang FC, Delobelle P, Nicol E. Explaining the impact of mHealth on maternal and child health care in low- and middle-income countries: a realist synthesis. BMC Pregnancy Childbirth. 2021;21(1):196. doi: 10.1186/s12884-021-03684-x 33750340 PMC7941738

[31] Almuslim H, AlDossary S. Models of Incorporating Telehealth into Obstetric Care During the COVID-19 Pandemic, Its Benefits And Barriers: A Scoping Review. Telemed J E Health. 2022;28(1):24–38. doi: 10.1089/tmj.2020.0553 33819434

[32] Vassilev I, Rowsell A, Pope C, Kennedy A, O’Cathain A, Salisbury C, et al. Assessing the implementability of telehealth interventions for self-management support: a realist review. Implement Sci. 2015;10:59. doi: 10.1186/s13012-015-0238-9 25906822 PMC4424965

[33] Greenhalgh T, Rosen R, Shaw SE, Byng R, Faulkner S, Finlay T, et al. Planning and Evaluating Remote Consultation Services: A New Conceptual Framework Incorporating Complexity and Practical Ethics. Front Digit Health. 2021;3:726095. doi: 10.3389/fdgth.2021.726095 34713199 PMC8521880

[34] CQC. Maternity survey 2023. 2023. Available from: www.cqc.org.uk

[35] Thomson G, Downe S. Emotions and support needs following a distressing birth: Scoping study with pregnant multigravida women in North-West England. Midwifery. 2016;40:32–9. doi: 10.1016/j.midw.2016.06.010 27428096

[36] Thomson G, Garrett C. Afterbirth support provision for women following a traumatic/distressing birth: Survey of NHS hospital trusts in England. Midwifery. 2019;71:63–70. doi: 10.1016/j.midw.2019.01.004 30690201

[37] Sharma A, Minh Duc NT, Luu Lam Thang T, Nam NH, Ng SJ, Abbas KS, et al. A Consensus-Based Checklist for Reporting of Survey Studies (CROSS). J Gen Intern Med. 2021;36(10):3179–87. doi: 10.1007/s11606-021-06737-1 33886027 PMC8481359

[38] Greenhalgh T, Ladds E, Hughes G, Moore L, Wherton J, Shaw SE, et al. Why do GPs rarely do video consultations? Qualitative study in UK general practice. British J General Practice. 2022:BJGP.2021.0658. 10.3399/BJGP.2021.0658 PMC8936181 35256385

[39] Tavener CR, Kyriacou C, Elmascri I, Cruickshank A, Das S. Rapid introduction of virtual consultation in a hospital-based Consultant-led Antenatal Clinic to minimise exposure of pregnant women to COVID-19. BMJ Open Qual. 2022;11(1):e001622. doi: 10.1136/bmjoq-2021-001622 35027342 PMC8761597

[40] NHS Digital. Digital Maternity: Harnessing Digital Technology in Maternity Services. 2021. Contract No.: 09/02/21.

[41] NHS England. Three year delivery plan for maternity and neonatal services. 2023.

[42] Darzi A. Independent investigation of the National Health Service in England. Department of Health and Social Care; 2024.

[43] RCOG. Maternity Triage: Good Practice Paper No. 17. December 2023.

[44] Gomez-Roas MV, Davis KM, Leziak K, Jackson J, Williams BR, Feinglass JM, et al. Postpartum during a pandemic: Challenges of low-income individuals with healthcare interactions during COVID-19. PLoS One. 2022;17(5):e0268698. doi: 10.1371/journal.pone.0268698 35609090 PMC9129029

[45] Spiby H, Faucher MA, Sands G, Roberts J, Kennedy HP. A qualitative study of midwives’ perceptions on using video-calling in early labor. Birth. 2019;46(1):105–12. doi: 10.1111/birt.12364 29901231

[46] Bailey CM, Newton JM, Hall HG. Telephone triage in midwifery practice: A cross-sectional survey. Int J Nurs Stud. 2019;91:110–8. doi: 10.1016/j.ijnurstu.2018.11.009 30682631

[47] Baron AM, Ridgeway JL, Stirn SL, Morris MA, Branda ME, Inselman JW, et al. Increasing the Connectivity and Autonomy of RNs with Low-Risk Obstetric Patients: Findings of a study exploring the use of a new prenatal care model. Am J Nurs. 2018;118(1):48–55. doi: 10.1097/01.NAJ.0000529715.93343.b0 29280806

[48] Greenhalgh T, Payne R, Hemmings N, Leach H, Hanson I, Khan A, et al. Training needs for staff providing remote services in general practice: a mixed-methods study. Br J Gen Pract. 2023;74(738):e17–26. doi: 10.3399/BJGP.2023.0251 38154935 PMC10756003

[49] Quinn LM, Olajide O, Green M, Sayed H, Ansar H. Patient and Professional Experiences With Virtual Antenatal Clinics During the COVID-19 Pandemic in a UK Tertiary Obstetric Hospital: Questionnaire Study. J Med Internet Res. 2021;23(8):e25549. doi: 10.2196/25549 34254940 PMC8409501

[50] Evans C, Spiby H, Barrett V, Clancy G, Sunney C. ARM@DA e-learning: How to provide safe, appropriate and acceptable digital consultations in maternity care. University of Nottingham; 2024. Available from: https://www.nottingham.ac.uk/helmopen/rlos/practice-learning/midwifery/telehealth/armada/index.html

[51] The Good Things Foundation. Dorset: Digital inclusion for those who are excluded: Maternity Matters. n.d.

[52] Mum and Baby App and Website. Mum and Baby: NHS Advice and Information for Pregnancy, Birth and Beyond. 2022. Available from: https://mumandbaby.uk/

[53] JANAM APP. One stop pregnancy information guide for South Asian mums. 2023. Available from: https://janamapp.co.uk/

[54] Cook JV, Dickinson HO, Eccles MP. Response rates in postal surveys of healthcare professionals between 1996 and 2005: an observational study. BMC Health Serv Res. 2009;9:160. doi: 10.1186/1472-6963-9-160 19751504 PMC2758861

[55] Timmins F, Ottonello G, Napolitano F, Musio ME, Calzolari M, Gammone M, et al. The state of the science-the impact of declining response rates by nurses in nursing research projects. J Clin Nurs. 2023;32(7–8):e9–11. doi: 10.1111/jocn.16597 36460485

