# Supplementary material for: A mapping survey of digital clinical consultations in maternity care in England
Source: PLOS Digit Health. 2025 Jul 21;4(7):e0000944. doi: 10.1371/journal.pdig.0000944 (PMC12279142; doi:10.1371/journal.pdig.0000944)
Supplement: S1 File — (PDF) [file pdig.0000944.s001.pdf]

Survey on digital consultations in maternity care

* Required

Introduction

Thank you for your interest in this survey exploring the use of digital (remote/virtual) consultations in maternity care in England. Before proceeding, please note the eligibility criteria for this survey:

- **Senior maternity professionals** and **digital maternity professionals** working in the NHS in England.
- E.g. directors/heads of midwifery or obstetrics, consultant midwives, matrons, digital midwives, digital leaders in midwifery or obstetrics.

Before starting the survey, please read the participant information sheet (https://armada-project.co.uk/wp-content/uploads/2024/01/participant-information-sheet.pdf) which will which give you more information about the survey and what will happen to the data you provide. It should take about 5 minutes to read.

If you have any questions about the survey and your participation the contact details for the research team can be found below and in the participant information sheet.

Principal investigator: Professor Catrin Evans, catrin.evans@nottingham.ac.uk

Lead researcher: Dr Georgia Clancy, georgia.clancy@nottingham.ac.uk

On the following page you will be asked to provide your consent to take part in the survey.

The survey itself will then take approximately 15 minutes to complete.

Consent form

1

I confirm that I have read and understood the information on the previous page, I have had the opportunity to consider the information, ask questions and have had these answered satisfactorily.

I am 18 years old and/or older

I understand that my participation is voluntary and I can end the survey at any time and withdraw my data by exiting the survey or closing the browser.

I understand that once the survey has been submitted it will not be possible to withdraw my participation.

I understand that the information I provide in this survey will be made anonymous before it is stored. It will be uploaded into a secure database on a computer kept in a secure place. Data will be kept for 7 years after the study has ended and then deleted.

I understand that the anonymised data from this study may be used in the future for research and teaching purposes.

By proceeding with this online survey I agree to the points above and agree to take part in this study.
*

◯ Yes

◯ No

Introductory questions

In this first block of questions please provide some brief information about your role and the NHS Trust in which you work. Please answer to the best of your knowledge.

2

What is your role/position? (if ‘other’ please specify)

- ◯ Director/Head of midwifery
- ◯ Midwifery matron
- ◯ Midwifery manager
- ◯ Digital midwife
- ◯ Consultant midwife
- ◯ Specialist midwife
- ◯ Head of obstetrics
- ◯ Consultant obstetrician
- ◯ Consultant obstetrician and gynaecologist
- ◯ Digital lead for obstetrics
- ◯ Other

3

What is your specific job title?

4

What NHS Trust do you work in (please write the full name without abbreviations)? - *please note that we are only collecting this information to keep track of which Trusts have completed the survey and it will not be reported further.*

5

Please tell us about the configuration of the maternity service in which you work:

- ◯ Single site
- ◯ Multiple site

6

If relevant, approximately, how many births does your maternity unit support per year?

- ◯ Less than 2,500
- ◯ 2,500 - 4,000
- ◯ 4,000 - 5,000
- ◯ 5,000 - 6,000
- ◯ More than 6,000

7

In what region of England is your NHS Trust based?

- ◯ South East England
- ◯ East Midlands
- ◯ North West
- ◯ North East
- ◯ Yorkshire and the Humber
- ◯ East of England
- ◯ West Midlands
- ◯ South West England
- ◯ Greater London

Digital consultation questions

We understand that the organisation of maternity care services varies between professions and from place to place. As such, please answer the following questions only on behalf of your own professional group, and in relation to your own areas of clinical responsibility in the place(s) where you work. If you feel that a question is not relevant to you, please move on to the next question.

In the questions below we have used the term **'clinical area'** to refer to the specific maternity setting in which you usually work or are responsible for e.g. labour ward, alongside maternity unit, freestanding maternity unit, community/home birth team.

When we refer to **digital consultations**, we mean: Synchronous **telephone** or **video** consultations involving direct interaction between a service user and a maternity healthcare professional. It has two-way functionality and can be initiated by either party. It may be linked to, or complemented by, other digital technologies within the maternity care pathways.

8

For **triage/maternity advice**, what methods of digital (i.e. virtual/remote) care does your professional group offer? *(If this question is not relevant to you please move on to the next question)*

	Yes	No	Unsure
Telephone call	◯	◯	◯
Video call (e.g. Attend Anywhere, Zoom, FaceTime)	◯	◯	◯
Messaging services (e.g. Text/SMS, WhatsApp)	◯	◯	◯
Email	◯	◯	◯
Apps (e.g. Badger Notes app)	◯	◯	◯
None, all appointments are in-person only	◯	◯	◯

9

If you use any other digital methods not listed above for **triage/maternity advice**, please tell us what they are:

[ ]

10

For the **antenatal booking visit**, what methods of digital care does your professional group offer?
(If this question is not relevant to you please move on to the next question)

	Yes	No	Unsure
Telephone call	◯	◯	◯
Video call (e.g. Attend Anywhere, Zoom, FaceTime)	◯	◯	◯
Messaging services (e.g. Text/SMS, WhatsApp)	◯	◯	◯
Email	◯	◯	◯
Apps (e.g. Badger Notes app)	◯	◯	◯
None, all booking appointments are in-person only	◯	◯	◯

11

If you use any other digital methods not listed above for the **antenatal booking visit**, please tell us what they are:

12

In the **antenatal community setting** (excluding booking visits), what methods of digital care does your professional group offer? *(If this question is not relevant to you please move on to the next question)*

	Yes	No	Unsure
Telephone call	◯	◯	◯
Video call (e.g. Attend Anywhere, Zoom, FaceTime)	◯	◯	◯
Messaging services (e.g. Text/SMS, WhatsApp)	◯	◯	◯
Email	◯	◯	◯
Apps (e.g. Badger Notes app)	◯	◯	◯
None, all appointments are in-person only	◯	◯	◯

13

If you use any other digital methods not listed above in the **antenatal community setting** (excluding booking visits), please tell us what they are:

14

In **antenatal hospital clinics**, what methods of digital care does your professional group offer? *(If this question is not relevant to you please move on to the next question)*

	Yes	No	Unsure
Telephone call	◯	◯	◯
Video call (e.g. Attend Anywhere, Zoom, FaceTime)	◯	◯	◯
Messaging services (e.g. Text/SMS, WhatsApp)	◯	◯	◯
Email	◯	◯	◯
Apps (e.g. Badger Notes app)	◯	◯	◯
None, all appointments are in-person only	◯	◯	◯

15

If you use any other digital methods not listed above in **antenatal hospital clinics,** please tell us what they are:

16

In **intrapartum hospital settings**, what methods of digital care does your professional group offer? *(If this question is not relevant to you please move on to the next question)*

	Yes	No	Unsure
Telephone call	○	○	○
Video call (e.g. Attend Anywhere, Zoom, FaceTime)	○	○	○
Messaging services (e.g. Text/SMS, WhatsApp)	○	○	○
Email	○	○	○
Apps (e.g. Badger Notes app)	○	○	○
None, all appointments are in-person only	○	○	○

17

If you use any other digital methods not listed above in **intrapartum hospital settings**, please tell us what they are:

18

In the **postnatal community setting**, what methods of digital care does your professional group offer? *(If this question is not relevant to you please move on to the next question)*

	Yes	No	Unsure
Telephone call	◯	◯	◯
Video call (e.g. Attend Anywhere, Zoom, FaceTime)	◯	◯	◯
Messaging services (e.g. Text/SMS, WhatsApp)	◯	◯	◯
Email	◯	◯	◯
Apps (e.g. Badger Notes app)	◯	◯	◯
None, all appointments are in-person only	◯	◯	◯

19

If you use any other digital methods not listed above in the **postnatal community setting**, please tell us what they are:

20

Are digital methods/technologies currently used for consultations in any other way not captured by the tables above? (this might include planned future use)

Digital consultation questions continued....

21

Please tell us what software or digital systems your clinical area uses for digital consultations (telephone and video calls) and why these were chosen:

22

Does your maternity service have a digital strategy?

- ◯ Yes
- ◯ No
- ◯ Don't know

23

If your maternity service does have a digital strategy, what organisation developed this?

24

What aspects of digital consultations does your organisation provide formal training on? (select all that apply, if 'other' please specify)

- ☐ No training provided
- ☐ Technical (e.g. using the software/technology)
- ☐ Information governance
- ☐ Incorporating digital/remote consultations into practice (e.g. time management, working from home etc.)
- ☐ Clinical need, assessing safety/risk
- ☐ Effective communication
- ☐ Personalisation of care
- ☐ Other

25

Does your clinical area have maternity specific guidelines/protocols for conducting digital consultations (telephone and video calls)?

- Yes
- No
- Don't know

26

If your clinical area does have maternity specific guidelines/protocols, what organisation produced these?

27

Who in your clinical area delivers digital consultations (telephone and video calls)? (select all that apply, if 'other' please specify)

- All midwives
- All obstetricians
- Some midwives
- Some obstetricians
- Don't know
- Other

28

In your clinical area are digital consultations (telephone and video calls) conducted as standalone digital clinics or are they integrated with face-to-face care? (If 'other' please specify)

- Standalone digital clinics
- Integrated with face-to-face care
- Don't know
- Other

29

Does your clinical area record how many consultations take place digitally (via telephone and video calls)?

- ◯ Yes
- ◯ No
- ◯ Don't know

30

Have staff in your clinical area been asked about their preferences for providing digital consultations? (If ‘other’ please specify)

- ◯ Yes
- ◯ No
- ◯ Don't know
- ◯ Other

31

Can staff opt-out of providing digital consultations if they wish? (If ‘other’ please specify)

- ◯ Yes
- ◯ No
- ◯ Don't know
- ◯ Other

Digital resources and connectivity

32

What digital devices do staff have access to for conducting digital consultations **on NHS sites**? (select all that apply)
(If this question is not relevant to you, please move on)

- ☐ Desktop computer (employer provided)
- ☐ Laptop computer (employer provided)
- ☐ Tablet (employer provided)
- ☐ Landline phone (employer provided)
- ☐ Mobile phone (employer provided)
- ☐ Personal digital devices
- ☐ It varies

33

What digital devices do staff have access to for conducting digital consultations **offsite/ community**? (select all the apply)
(If this question is not relevant to you, please move on)

- ☐ Desktop computer (employer provided)
- ☐ Laptop computer (employer provided)
- ☐ Tablet (employer provided)
- ☐ Landline phone (employer provided)
- ☐ Mobile phone (employer provided)
- ☐ Personal digital devices
- ☐ It varies

34

Do staff have digital connectivity (e.g. WiFi/internet, mobile data allowance) with which to conduct digital consultations **on NHS sites** and/or **offsite**? (select all that apply, if 'other' please specify)

- ☐ Staff have digital connectivity on NHS sites and offsite
- ☐ Staff have digital connectivity on NHS sites only
- ☐ Staff are given digital connectivity resources (e.g. WiFi dongle) for offsite work
- ☐ Staff use personal internet/data allowances
- ☐ Other

35

How suitable are the **digital devices** (e.g. laptop, mobile phone) and **digital connectivity resources** (e.g. WiFi, mobile data) that staff have for conducting digital consultations **on NHS sites** and **offsite**?

	Very poor	Poor	Acceptable	Good	Very go
Digital devices - *on NHS sites*	○	○	○	○	○
Digital devices - *offsite*	○	○	○	○	○
Digital connectivity - *on NHS sites*	○	○	○	○	○
Digital connectivity - *offsite*	○	○	○	○	○

36

When staff conduct digital consultations (telephone and video calls) **on NHS sites**, do they have dedicated spaces in which to conduct their consultations (i.e. somewhere private and quiet)?
(If 'other' please specify)

- ○ Yes
- ○ No
- ○ Don't know
- ○ Other

Women and service users

37

Are women/service users asked about their consultation preferences at any point during their maternity care? I.e. if they are happy to have digital consultations and by what technological modality?

- ◯ Yes - formally recorded in notes
- ◯ Yes - informally, *not* recorded in notes
- ◯ No
- ◯ Don't know

38

Are consultation preferences regularly reviewed with women/service users throughout their maternity care journey?

- ◯ Yes
- ◯ No
- ◯ Don't know

39

What aspects of women's suitability for digital consultations are assessed? (select all that apply, if 'other' please specify)

- ☐ No assessment
- ☐ Clinical need/risk (including when an in-person assessment is needed)
- ☐ Access to digital devices/connectivity and digital literacy
- ☐ Language/communication needs
- ☐ Psychosocial status (e.g. mental health conditions)
- ☐ Safeguarding concerns
- ☐ Other

40

Are women provided with information/support to help them access digital consultations? (select all that apply, if 'other' please specify)

- ☐ No information/support provided
- ☐ Yes - verbal information
- ☐ Yes - printed leaflets/information
- ☐ Yes - digital information
- ☐ Yes - videos
- ☐ Other

41

Does your organisation have policies/procedures in place for supporting equitable access to digital consultations for women/service users?

- ○ Yes
- ○ No
- ○ Don't know

42

If your organisation does have policies/procedures for supporting equitable access, what are they?

43

Does your clinical area offer access to translators, if necessary, for digital consultations? E.g. for those for whom English is not their first language or who have a hearing disability?

- ○ Yes
- ○ No
- ○ Don't know

Final thoughts...

44

Which parts of the maternity service do you think will continue to use digital consultations (telephone and video calls) in the future?

45

Does your clinical area collect satisfaction data specifically in relation to digital consultations (telephone and video calls)?

- ◯ Yes
- ◯ No
- ◯ Don't know

46

What, if any, concerns do you have about the use of digital consultations in maternity care?

47

What, if any, benefits have you noticed about using digital consultations in maternity care?

48

Do you have any other thoughts or comments about the use of digital consultations in maternity care which you would like to add?

49

Question

	Option 1	Option 2	Option 3	Option 4	Optio[illegible]
Statement 1	◯	◯	◯	◯	◯
Statement 2	◯	◯	◯	◯	◯
