# Supplementary material for: A mapping survey of digital clinical consultations in maternity care in England
Source: PLOS Digit Health. 2025 Jul 21;4(7):e0000944. doi: 10.1371/journal.pdig.0000944 (PMC12279142; doi:10.1371/journal.pdig.0000944)
Supplement: S3 File — (DOCX) [file pdig.0000944.s003.docx]

# Supporting Information File – Dataset

| **2. What is your role/position? (if ‘other’ please specify)** | no. |
| --- | --- |
| Director/Head of midwifery | 5 |
| Midwifery matron | 4 |
| Midwifery manager | 0 |
| Digital midwife | 25 |
| Consultant midwife | 5 |
| Specialist midwife | 3 |
| Head of obstetrics | 0 |
| Consultant obstetrician | 4 |
| Consultant obstetrician and gynaecologist | 5 |
| Digital lead for obstetrics | 2 |

| **3. What is your specific job title?** |
| --- |
| Not reported in paper for anonymity. |

| **4. What NHS Trust do you work in (please write the full name without abbreviations)?** |
| --- |
| Not reported in paper for anonymity. |

| **5. Please tell us about the configuration of the maternity service in which you work:** | no. |
| --- | --- |
| Single site | 27 |
| Multiple site | 26 |

| **6. If relevant, approximately, how many births does your maternity unit support per year?** | no. |
| --- | --- |
| Less than 2,500 | 4 |
| 2,500 - 4,000 | 12 |
| 4,000 - 5,000 | 13 |
| 5,000 - 6,000 | 7 |
| More than 6,000 | 17 |

| **7. In what region of England is your NHS Trust based?** | no. |
| --- | --- |
| South East England | 5 |
| East Midlands | 4 |
| North West | 5 |
| North East | 3 |
| Yorkshire and the Humber | 4 |
| East of England | 13 |
| West Midlands | 8 |
| South West England | 3 |
| Greater London | 8 |

| **8. For triage/maternity advice, what methods of digital (i.e. virtual/remote) care does your professional group offer?** | Yes | No | Unsure | Blank/ unanswered |
| --- | --- | --- | --- | --- |
| Telephone call | 52 | 1 | 0 | 0 |
| Video call (e.g. Attend Anywhere, Zoom, FaceTime) | 44 | 2 | 2 | 5 |
| Messaging services (e.g. Text/SMS, WhatsApp) | 9 | 39 |  | 5 |
| Email | 11 | 35 | 2 | 5 |
| Apps (e.g. Badger Notes app) | 18 | 28 | 1 | 6 |
| None, all appointments are in-person only | 7 | 31 | 1 | 14 |

| **9. If you use any other digital methods not listed above for triage/maternity advice, please tell us what they are:** |
| --- |
| Not reported in paper. |

| **10. For the antenatal booking visit, what methods of digital care does your professional group offer?** | Yes | No | Unsure | Blank/ unanswered |
| --- | --- | --- | --- | --- |
| Telephone call | 19 | 26 | 1 | 7 |
| Video call (e.g. Attend Anywhere, Zoom, FaceTime) | 6 | 38 | 0 | 9 |
| Messaging services (e.g. Text/SMS, WhatsApp) | 3 | 41 | 0 | 9 |
| Email | 2 | 40 | 1 | 10 |
| Apps (e.g. Badger Notes app) | 9 | 34 | 1 | 9 |
| None, all appointments are in-person only | 37 | 10 | 0 | 6 |

| **11. If you use any other digital methods not listed above for the antenatal booking visit, please tell us what they are:** |
| --- |
| Not reported in paper. |

| **12. In the antenatal community setting (excluding booking visits), what methods of digital care does your professional group offer?** | Yes | No | Unsure | Blank/ unanswered |
| --- | --- | --- | --- | --- |
| Telephone call | 33 | 12 | 2 | 6 |
| Video call (e.g. Attend Anywhere, Zoom, FaceTime) | 3 | 35 | 1 | 14 |
| Messaging services (e.g. Text/SMS, WhatsApp) | 14 | 24 | 3 | 12 |
| Email | 10 | 27 | 2 | 14 |
| Apps (e.g. Badger Notes app) | 13 | 29 | 1 | 10 |
| None, all appointments are in-person only | 27 | 16 | 2 | 8 |

| **13. If you use any other digital methods not listed above in the antenatal community setting (excluding booking visits), please tell us what they are:** |
| --- |
| Not reported in paper. |

| **14. In antenatal hospital clinics, what methods of digital care does your professional group offer?** | Yes | No | Unsure | Blank/ unanswered |
| --- | --- | --- | --- | --- |
| Telephone call | 38 | 8 | 1 | 6 |
| Video call (e.g. Attend Anywhere, Zoom, FaceTime) | 12 | 26 | 2 | 13 |
| Messaging services (e.g. Text/SMS, WhatsApp) | 7 | 33 | 1 | 12 |
| Email | 7 | 33 | 0 | 13 |
| Apps (e.g. Badger Notes app) | 12 | 29 | 1 | 11 |
| None, all appointments are in-person only | 23 | 21 | 0 | 9 |

| **15. If you use any other digital methods not listed above in antenatal hospital clinics, please tell us what they are:** |
| --- |
| Not reported in paper. |

| **16. In intrapartum hospital settings, what methods of digital care does your professional group offer?** | Yes | No | Unsure | Blank/ unanswered |
| --- | --- | --- | --- | --- |
| Telephone call | 15 | 25 | 0 | 13 |
| Video call (e.g. Attend Anywhere, Zoom, FaceTime) | 1 | 35 | 0 | 17 |
| Messaging services (e.g. Text/SMS, WhatsApp) | 0 | 36 | 0 | 17 |
| Email | 0 | 35 | 0 | 18 |
| Apps (e.g. Badger Notes app) | 4 | 32 | 0 | 17 |
| None, all appointments are in-person only | 45 | 4 | 0 | 4 |

| **17. If you use any other digital methods not listed above in intrapartum hospital settings, please tell us what they are:** |
| --- |
| Not reported in paper. |

| **18. In the postnatal community setting, what methods of digital care does your professional group offer?** | Yes | No | Unsure | Blank/ unanswered |
| --- | --- | --- | --- | --- |
| Telephone call | 39 | 5 | 2 | 7 |
| Video call (e.g. Attend Anywhere, Zoom, FaceTime) | 2 | 33 | 2 | 16 |
| Messaging services (e.g. Text/SMS, WhatsApp) | 15 | 23 | 3 | 12 |
| Email | 4 | 35 | 1 | 13 |
| Apps (e.g. Badger Notes app) | 12 | 29 | 1 | 11 |
| None, all appointments are in-person only | 21 | 18 | 2 | 12 |

| **19. If you use any other digital methods not listed above in the postnatal community setting, please tell us what they are:** |
| --- |
| Not reported in paper. |

| **20. Are digital methods/technologies currently used for consultations in any other way not captured by the tables above? (this might include planned future use)** |
| --- |
| Not reported in paper. |

| **21. Please tell us what software or digital systems your clinical area uses for digital consultations (telephone and video calls) and why these were chosen:** |
| --- |
| Not reported in paper. |

| **22. Does your maternity service have a digital strategy?** | no. |
| --- | --- |
| Yes | 50 |
| No | 0 |
| Don't know | 3 |

| **23. If your maternity service does have a digital strategy, what organisation developed this?** | no. |
| --- | --- |
| ‘In-house’ | 9 |
| Local Maternity and Newborn System(s) | 9 |
| NHS Trust(s) | 8 |
| Digital midwife | 6 |
| Don’t know | 4 |
| NHS | 3 |
| Software company | 1 |
| N/A | 1 |

| **24. What aspects of digital consultations does your organisation provide formal training on? (select all that apply, if ‘other’ please specify)** | no. |
| --- | --- |
| No training provided | 11 |
| Technical (e.g. using the software/technology) | 25 |
| Information governance | 32 |
| Incorporating digital/remote consultations into practice (e.g. time management, working from home etc.) | 3 |
| Clinical need, assessing safety/risk | 15 |
| Effective communication | 10 |
| Personalisation of care | 14 |
| Other | 1 |

| **25. Does your clinical area have maternity specific guidelines/protocols for conducting digital consultations (telephone and video calls)?** | no. |
| --- | --- |
| Yes | 5 |
| No | 30 |
| Don't know | 18 |

| **26. If your clinical area does have maternity specific guidelines/protocols, what organisation produced these?** |
| --- |
| Not reported in paper. |

| **27. Who in your clinical area delivers digital consultations (telephone and video calls)?** | no. |
| --- | --- |
| All midwives | 3 |
| All obstetricians | 8 |
| Some midwives | 39 |
| Some obstetricians | 35 |
| Don't know | 1 |
| Other | 10 |

| **28. In your clinical area are digital consultations (telephone and video calls) conducted as standalone digital clinics or are they integrated with face-to-face care?** | no. |
| --- | --- |
| Standalone digital clinics | 1 |
| Integrated with face-to-face care | 42 |
| Don't know | 2 |
| Other | 7 |
|  |  |
| **29. Does your clinical area record how many consultations take place digitally (via telephone and video calls)?** | no. |
| Yes | 21 |
| No | 15 |
| Don't know | 15 |
|  |  |
| **30. Have staff in your clinical area been asked about their preferences for providing digital consultations?** | no. |
| Yes | 7 |
| No | 25 |
| Don't know | 20 |
| Other | 1 |
|  |  |
| **31. Can staff opt-out of providing digital consultations if they wish?** | no. |
| Yes | 15 |
| No | 15 |
| Don't know | 19 |
| Other | 4 |

| **32. What digital devices do staff have access to for conducting digital consultations on NHS sites?** | no. |
| --- | --- |
| Desktop computer (employer provided) | 47 |
| Laptop computer (employer provided) | 38 |
| Tablet (employer provided) | 11 |
| Landline phone (employer provided) | 44 |
| Mobile phone (employer provided) | 28 |
| Personal digital devices | 6 |
| It varies | 10 |
|  |  |
| **33. What digital devices do staff have access to for conducting digital consultations offsite/ community?** | no. |
| Desktop computer (employer provided) | 17 |
| Laptop computer (employer provided) | 41 |
| Tablet (employer provided) | 11 |
| Landline phone (employer provided) | 15 |
| Mobile phone (employer provided) | 41 |
| Personal digital devices | 6 |
| It varies | 10 |
|  |  |
| **34. Do staff have digital connectivity (e.g. Wi-Fi/internet, mobile data allowance) with which to conduct digital consultations on NHS sites and/or offsite?** | no. |
| Staff have digital connectivity on NHS sites and offsite | 37 |
| Staff have digital connectivity on NHS sites only | 10 |
| Staff are given digital connectivity resources for offsite work | 24 |
| Staff use personal internet/data allowances | 8 |
| Other | 7 |

| **35. How suitable are the digital devices (e.g. laptop, mobile phone) and digital connectivity resources (e.g. Wi-Fi, mobile data) that staff have for conducting digital consultations on NHS sites and offsite? %** | Very poor | Poor | Accept-able | Good | Very good | Blank/ unanswered |
| --- | --- | --- | --- | --- | --- | --- |
| Digital connectivity - offsite | 6 | 17 | 16 | 7 | 3 | 4 |
| Digital connectivity - on NHS sites | 0 | 2 | 10 | 23 | 16 | 2 |
| Digital devices - offsite | 1 | 4 | 17 | 19 | 7 | 5 |
| Digital devices - on NHS sites | 0 | 3 | 11 | 23 | 14 | 2 |

| **36. When staff conduct digital consultations (telephone and video calls) on NHS sites, do they have dedicated spaces in which to conduct their consultations?** | no. |
| --- | --- |
| Yes | 36 |
| No | 4 |
| Don't know | 3 |
| Other | 2 |
| It varies | 6 |

| **37. Are women/service users asked about their consultation preferences at any point during their maternity care? I.e. if they are happy to have digital consultations and by what technological modality?** | no. |
| --- | --- |
| Yes - formally recorded in notes | 7 |
| Yes - informally, not recorded in notes | 15 |
| No | 17 |
| Don't know | 14 |

| **38. Are consultation preferences regularly reviewed with women/service users throughout their maternity care journey?** | no. |
| --- | --- |
| Yes | 10 |
| No | 25 |
| Don't know | 17 |

| **39. What aspects of women’s suitability for digital consultations are assessed?** | no. |
| --- | --- |
| No assessment | 19 |
| Clinical need/risk (including when an in-person assessment is needed) | 26 |
| Access to digital devices/connectivity and digital literacy | 23 |
| Language/communication needs | 28 |
| Psychosocial status (e.g. mental health conditions) | 23 |
| Safeguarding concerns | 25 |
| Don't know | 1 |
| N/A | 3 |

| **40. Are women provided with information/support to help them access digital consultations?** | no. |
| --- | --- |
| No information/support provided | 22 |
| Yes - verbal information | 17 |
| Yes - printed leaflets/information | 10 |
| Yes - digital information | 7 |
| Yes - videos | 3 |
| Don't know | 2 |
| N/A | 4 |

| **41. Does your organisation have policies/procedures in place for supporting equitable access to digital consultations for women/service users?** | no. |
| --- | --- |
| Yes | 7 |
| No | 17 |
| Don't know | 28 |

| **42. If your clinical area does have maternity specific guidelines/protocols, what organisation produced these?** | |
| --- | --- |
| Illustrative excerpts: | *Interpreters, translated resources, phones for text, digital gifting, data bank.* |

| **43. Does your clinical area offer access to translators, if necessary, for digital consultations? E.g. for those for whom English is not their first language or who have a hearing disability?** | no. |
| --- | --- |
| Yes | 48 |
| No | 0 |
| Don't know | 5 |

| **44. Which parts of the maternity service do you think will continue to use digital consultations (telephone and video calls) in the future?** |
| --- |
| Not reported in paper. |

| **45. Does your clinical area collect satisfaction data specifically in relation to digital consultations (telephone and video calls)?** | no. |
| --- | --- |
| Yes | 3 |
| No | 36 |
| Don't know | 11 |

| **46, 47, 48. Concerns about the use of digital consultations in maternity care.** | no. |
| --- | --- |
| Digital literacy | 12 |
| Lack of physical examination/tests | 8 |
| Communication barriers | 7 |
| Digital readiness | 6 |
| Safety (including safeguarding) | 6 |
| Perceived to not meet service user preferences | 4 |
| Poor digital systems | 4 |
| Lack of training/guidance | 3 |
| Little/no perceived benefits | 2 |
| Use of AI | 1 |

| **46, 47, 48. Benefits of using digital consultations in maternity care.** | no. |
| --- | --- |
| Reduces service user burden of care | 25 |
| Increased flexibility/efficiency for providers | 8 |
| Provides an alternative to in-person care | 7 |
| Meets service user preferences | 4 |
| Supports at-home monitoring | 2 |
| Supports triage/initial assessment | 2 |
